# Deep learning-based Emergency Department In-hospital Cardiac Arrest Score (Deep EDICAS) for early prediction of cardiac arrest and cardiopulmonary resuscitation in the emergency department

**DOI:** 10.1186/s13040-024-00407-8

**Published:** 2024-11-23

**Authors:** Yuan-Xiang Deng, Jyun-Yi Wang, Chia-Hsin Ko, Chien-Hua Huang, Chu-Lin Tsai, Li-Chen Fu

**Affiliations:** 1 Department of Computer Science and Information Engineering, National Taiwan University, CSIE Der Tian Hall No. 1, Sec. 4, Roosevelt Road, Taipei, 106319 Taiwan; 2 Department of Emergency Medicine, National Taiwan University College of Medicine and National Taiwan University Hospital, 7 Zhongshan S. Rd, Taipei, 100225 Taiwan

**Keywords:** Emergency department, Cardiac arrest, Early warning system, Multi-modal model, Time series data

## Abstract

**Background:**

Timely identification of deteriorating patients is crucial to prevent the progression to cardiac arrest. However, current methods predicting emergency department cardiac arrest are primarily static, rule-based with limited precision and cannot accommodate time-series data. Deep learning has the potential to continuously update data and provide more precise predictions throughout the emergency department stay.

**Methods:**

We developed and internally validated a deep learning-based scoring system, the Deep EDICAS for early prediction of cardiac arrest and a subset of arrest, cardiopulmonary resuscitation (CPR), in the emergency department. Our proposed model effectively integrates tabular and time series data to enhance predictive accuracy. To address data imbalance and bolster early prediction capabilities, we implemented data augmentation techniques.

**Results:**

Our system achieved an AUPRC of 0.5178 and an AUROC of 0.9388 on on data from the National Taiwan University Hospital. For early prediction, our system achieved an AUPRC of 0.2798 and an AUROC of 0.9046, demonstrating superiority over other early warning scores. Moerover, Deep EDICAS offers interpretability through feature importance analysis.

**Conclusion:**

Our study demonstrates the effectiveness of deep learning in predicting cardiac arrest in emergency department. Despite the higher clinical value associated with detecting patients requiring CPR, there is a scarcity of literature utilizing deep learning in CPR detection tasks. Therefore, this study embarks on an initial exploration into the task of CPR detection.

## Background

The pervasive global problem of Emergency Department (ED) overcrowding in a hospital leads to significant consequences, such as rising patient numbers, increased mortality and morbidity rates, and a compromised ability to deliver crucial services to patients facing medical emergencies promptly. Recent studies have focused on understanding the causes and impacts of ED congestion [1–3]. Due to ED overcrowding, inadequate healthcare staffing and unstable patient conditions, incidents of IHCA (In-Hospital Cardiac Arrest) are more likely to occur in the ED. IHCA refers to the sudden cessation of cardiac pumping function within a hospital setting. According to data from the American Heart Association’s Resuscitation Registry, the incidence rate of IHCA in hospitalized adults is approximately 10 cases per thousand bed-days( 290,000 patients per year), about 10% of which occurred in the emergency department (ED), with only 25% to 40% of adult IHCA patients surviving to discharge [4].

Timely identification of deteriorating patients is crucial to prevent the progression to cardiac arrest and increase the chances of successful resuscitation. Currently, in clinical practice, Early Warning Scores (EWS) serve as tools for assessing and scoring vital signs, enabling healthcare professionals to detect subtle changes in a patient’s condition before a critical event occurs. Representative EWS systems include the Modified Early Warning Score (MEWS) [5] and the National Early Warning Score (NEWS) [6]. We previously developed an EWS system for cardiac arrest, the Emergency Department In-hospital Cardiac Arrest Score (EDICAS) [7], specifically designed for IHCA detection in the ED (Table 1). We later also validated the EDICAS using a different ED patient population [8]. The EWS systems typically involve assessing of vital signs such as heart rate, respiratory rate, blood pressure, temperature, and other parameters. Each vital sign is assigned a score based on predetermined thresholds, contributing to determining of the patient’s risk level. When the EWS reaches specific thresholds, it prompts healthcare providers to assess the patient, initiate further investigations, or escalate care as needed. This proactive approach enables timely interventions, helping to prevent or mitigate the severity of adverse events like cardiac arrest and aids in resource allocation for healthcare staff.

**Table 1:** The items and scoring of the EDICAS

	Score		
Items	1	2	3
Age, year		$\ge$65	
Arrival by ambulance	Yes		
Systolic blood pressure, mm Hg			<90
Heart rate, beats per min	<60 or >90		
Body temperature, $^{\circ }$C		<36	
Respiratory rate, breaths per minute		$\ge$22	
Oxygen saturation, %	<95		
GCS <15 or acute change in levels of consciousness	Yes		

In 2018, a deep learning (DL)-based early warning system (DEWS) was developed [9]. The DEWS assessed the risk of cardiac arrest (CA) within 24 hours of observing vital signs on the ward. DEWS demonstrated potential in predicting CA, showing higher sensitivity and lower false alarm rates than the originally developed MEWS. There was a growing trend in applying DL to early warning systems. We aimed to develop and internally validate a DL-based scoring system, the Deep EDICAS, for early prediction of cardiac arrest and cardiopulmonary resuscitation in the ED.

### Motivation

Due to infrequent vital sign measurements and unstable patient conditions, CA events are more likely to occur unexpectedly in the emergency department. However, previous research on CA has mainly focused on general ward patients [5, 10], with limited studies specifically targeting CA prediction in the ED setting [11–13].

Research has shown that using a hybrid deep learning model (combining LSTM and MLP) improves MEWS (Modified Early Warning Score) and conventional machine learning algorithms in forecasting CA in ED [14]. This hybrid model, utilizing both tabular and time series data, achieved superior performance. However, it should be noted that this model relies on earlier-developed deep learning architectures. There is a scarcity of literature exploring deep learning for predicting cardiac arrests in emergency departments, leaving room for further refinement, including model selection and handling of data imbalance.

**Fig. 1:**
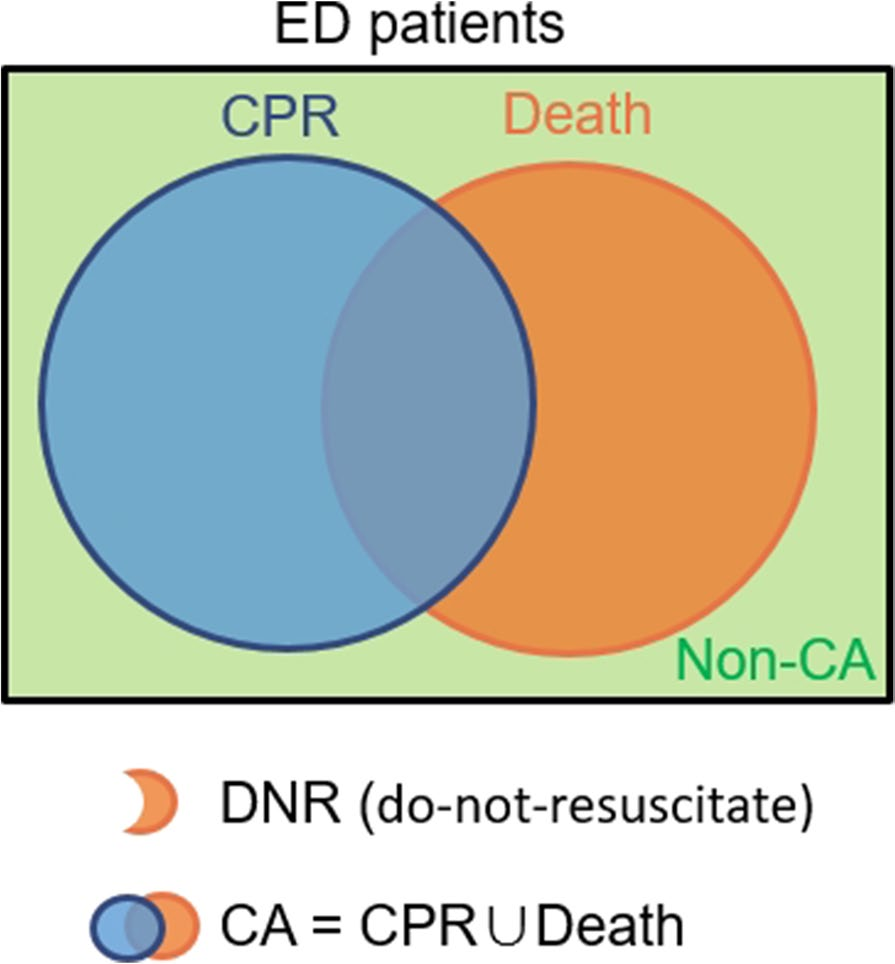
The Venn diagram of cardiac arrest. Cardiac arrest is a union of cardiopulmonary resuscitation and death. Abbreviations: CPR = cardiopulmonary resuscitation. CA = cardiac arrest. DNR = do not resuscitate

In this research work, we aim to develop a more accurate early warning system for patients with potential cardiac arrest in the ED and implement it in clinical practice. Early prediction helps to identify priority; providing more time for patient families to be prepared for the worse outcome and allowing healthcare professionals additional buffer time is crucial. Besides predicting CA, we also aim to predict a subset of CA, CPR (Cardiopulmonary Resuscitation). As illustrated in Fig. 1, some CA patients have already signed do-not-resuscitate (DNR) orders and hence do not require CPR. Identifying patients who require CPR early is rarely discussed in prior researches but holds significant clinical relevance, which we intend to incorporate in this study.

This research is of paramount importance in addressing in-hospital cardiac arrests in the emergency department, an understudied yet critical area. The immediate implementation of such an early warning response system might identify high-risk patients and prevent numerous in-hospital deaths. Our long-term goal is to deploy our system into existing clinical workflow in the National Taiwan University Hospital (NTUH) to assist healthcare professionals.

### Challenges

#### Irregular data’s sampling rates and frequent missing values

The measurement values of patients’ vital signs collected in the emergency room pose a challenge in applying deep learning models due to the substantial issue of missing values. Nurses measure a patient’s vital signs every few hours, occasionally resulting in long intervals between two adjacent instances. Moreover, these intervals vary significantly, and not all vital signs are measured during each instance. Consequently, dealing with irregular sampling rates and frequent missing values in our data format is challenging. Following the past research, we standardize the time intervals for the time series data to one hour. The actual missing value rate in our data is depicted in Table 2.

**Table 2:** The missing rate of vital signs

Vital sign	Missing rate
Systolic blood pressure	0.7180
Diastolic blood pressure	0.7180
Heart rate	0.7272
Body temperature	0.7341
Respiratory rate	0.7328
Oxygen saturation	0.8147

#### The data imbalance issue is severe

In addition to predicting cardiac arrest (CA), this study also undertakes the prediction of cardiopulmonary resuscitation (CPR). CA includes patients who experience death. Numerous studies have achieved good performance in death prediction [15, 16]. Predicting CPR becomes more challenging when excluding patients with more severe medical conditions. In our dataset, CPR constitutes 25% of CA cases, making the imbalance issue more severe for CPR than CA. The imbalance ratio for CA is 0.9%, whereas the imbalance ratio for CPR is 0.2%.

#### Efficient use of multi-modality data

Our dataset comprises both sequential data and tabular data. How to effectively leverage both data types simultaneously has been less discussed in the past literature, particularly as tabular data remains an area where deep learning has yet to make significant inroads [17].

#### Interpretability

In the medical domain, interpretability holds immense importance. A reliable medical diagnostic system should possess transparency, comprehensibility, and explainability to garner trust from physicians, supervisors, and patients. Ideally, it should provide reasoning behind its decisions to all stakeholders. Ensuring fairness and trustworthiness is paramount when implementing AI techniques or models in practical settings. Therefore, a data-driven model without interpretability might not be appropriate for making clinical decisions.

### Related works

In recent years, there have been attempts to employ various artificial neural network algorithms to predict deteriorating conditions (Table 3). Kwon et al. [9] developed a deep learning-based early warning system (DEWS) utilizing LSTM to identify patients with cardiac arrest (CA). Shamout et al. [18] crafted another DEWS employing bi-directional LSTM with an attention mechanism to forecast the likelihood of an adverse event, defined as the combined outcome of CA, mortality, or unplanned ICU admission. Apart from recurrent neural networks, studies exploring CA prediction have also delved into using TCN (Temporal Convolutional Network), a model leveraging a convolutional neural network (CNN) framework to handle time-series data [19].

**Table 3:** Comparison between related studies and our proposed work

	Task(s)	Data type	Deep learning	Utilizing static (s) and dynamic (d) feature	Explainable
EWS [5–7]	Adverse event	vital signs		s	$\checkmark$
Kwon et al. [9]	CA	Time series vital signs	$\checkmark$	d	
Shamout et al. [18]	CA	Time series vital signs	$\checkmark$	d	$\checkmark$
Tang et al. [19]	Adverse event (CA, mortality, unplanned ICU)	Time series vital signs	$\checkmark$	d	$\checkmark$
Jang et al. [14]	CA	Time series vital signs + Tabular data	$\checkmark$	s/d	
**Ours**	CA & CPR	Time series vital signs + Tabular data	$\checkmark$	s/d	$\checkmark$

In addition to temporal data encompassing vital signs, the patient data in the emergency department comprises tabular information involving demographics and triage details. Jang et al. [14] devised a hybrid deep learning model utilizing MLP to extract static features (tabular data) and LSTM to extract dynamic features (time-series data).

In contrast to previous studies, we introduce a more challenging CPR prediction task, designing a hybrid model that efficiently extracts features and offers interpretability. Additionally, we incorporated data augmentation to address the issue of data imbalance.

### Objectives

As mentioned earlier in this paper, we will encounter four challenges: missing values in the dataset, data imbalance, different data modalities, and model interpretability. In this section, we will establish four objectives corresponding to the challenges mentioned above.

#### Concerning missing values in the dataset

Considering that not every vital sign is measured during an instance, our designed model will handle univariate and multivariate dynamic features separately through encoders. We will provide more details regarding our model architecture in .

#### Concerning data imbalance

In this research work, we employ data augmentation to alleviate the issue of data imbalance. To address rare cases of CA in the emergency department, we aim to conduct data augmentation on CA samples, narrowing the gap between positive and negative sample quantities. The implementation of window shifting will assist the model in acquiring early prediction capabilities.

#### Concerning different data modalities

It is useful to develop develop a multi-modal model that can effectively utilize time series and tabular data to maximize the available information regarding emergency department patients for model learning, we will design a model capable of effectively utilizing tabular and time-series data. The two data input encoders will extract baseline and temporal features, followed by utilizing a multi-head self-attention mechanism to merge these two features effectively.

#### Concerning model interpretability

To apply deep learning in medical field, it becomes increasingly important to provide interpretability in the model’s predictions. While deep learning can achieve high performance, it is often seen as a black box due to the lack of insight into the model’s decision-making process. The traditional EWS (Early Warning Score) will explicitly reveal the vital signs which indicate signs of patient deterioration. Therefore, we aim to provide interpretability in our model to enable hospital professionals to understand better how our model makes decisions.

## Methods

### System overview

**Fig. 2:**
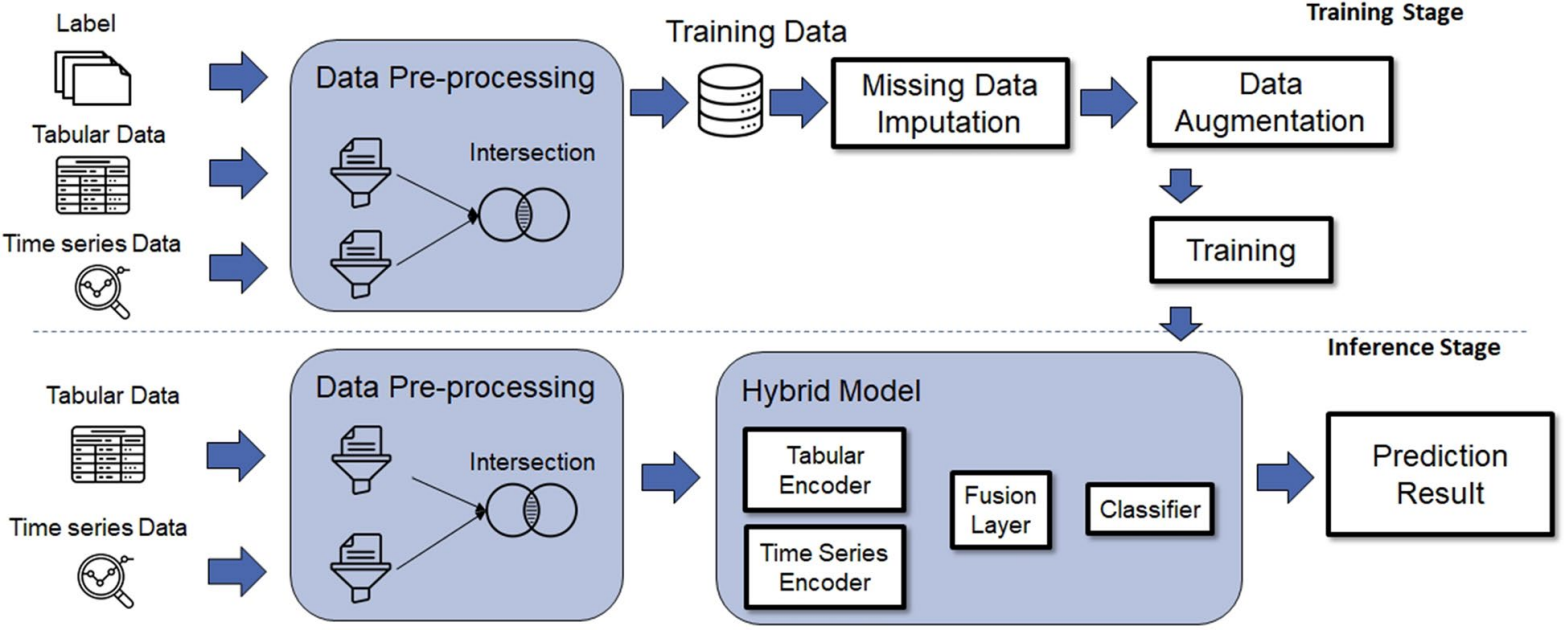
System overview. The upper part denotes the training state, and the lower part denotes the inference stage

The overview of the proposed early warning system is shown in Fig. 2. It can be divided into two parts: the training stage and the inference stage.

During the training stage, there are four essential components: data pre-processing, missing value imputation, data augmentation, and model training. Firstly, the raw data obtained from the database of the Emergency Department at National Taiwan University Hospital (NTUH), including tabular data, time series data, and labels, undergo data pre-processing and missing value imputation to obtain the set of training data for training the deep model. Next, data augmentation is performed on the minority class data to mitigate the problem of data imbalance and improve the model’s ability to make early predictions. Finally, the deep model is trained using the augmented data.

During the inference stage, the tabular and time series data undergo the same data pre-processing and missing value imputation methods as in the training stage. Finally, the model is available to predict the patients’ need for CPR (Cardiopulmonary Resuscitation). The detailed implementations of each part will be described in the following sections.

**Fig. 3:**
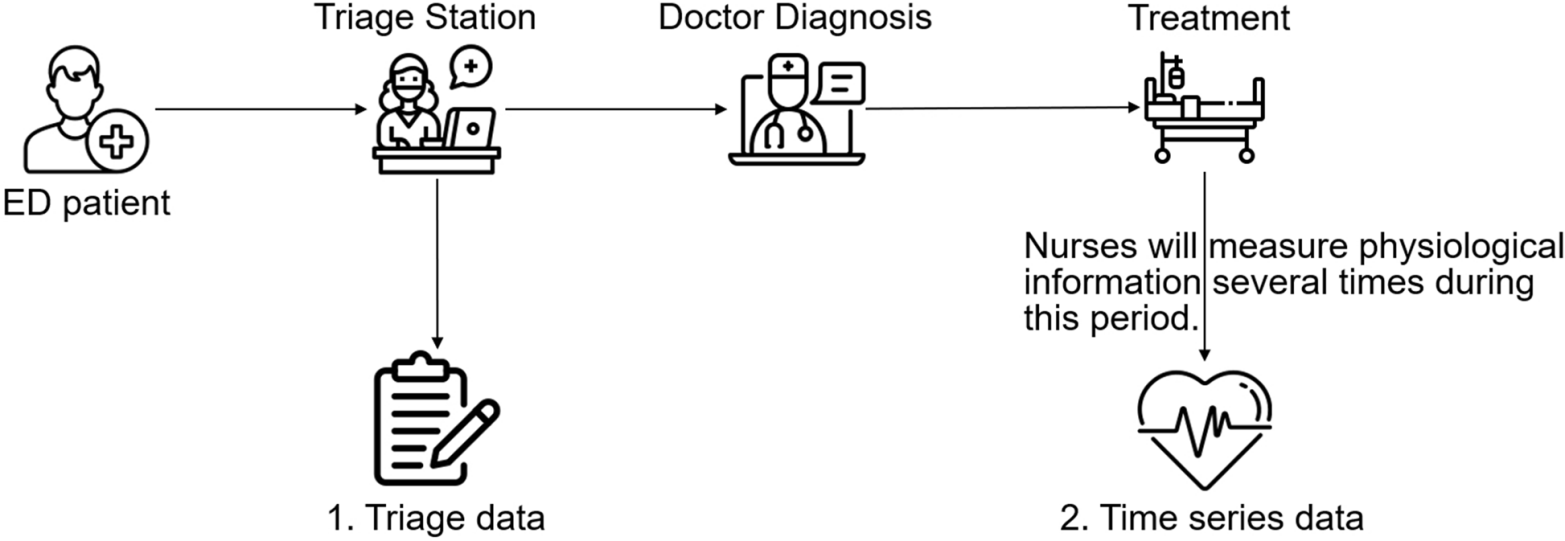
Data collection throughout the emergency department stay from triage to the observation area

### Data preparation

With the approval of the NTUH Institutional Review Board, we have conducted a retrospective study using data from the integrated Medical Database of NTUH. This database serves as the central clinical data repository for electronic health records within the healthcare system. We used data on ED visits for the main hospital between January 1, 2016 and December 31, 2019 for the current study.

The data collection process is depicted in Fig. 3. Upon arriving at the emergency department, patients first visit the triage station. Here, nurses assess the severity of the patient’s condition and assign a triage level to determine the treatment order. During triage, nurses gather basic patient information and measure vital signs recorded in the hospital’s electronic database. Subsequently, some patients may be admitted for further treatment after being diagnosed by emergency physicians. Throughout the treatment process, nurses irregularly measure the patient’s vital signs multiple times (depending on the patient’s condition) and manually record this information in the hospital’s electronic database.

#### Data pre-processing

Pre-processing steps are required to conform the data obtained from the database to the format suitable for input into the model. We will delineate these steps into two parts, one for handling tabular data and another for time series data.

##### Tabular data

The tabular data comprises the variables we have extracted from the triage data presented in Fig. 3. These variables can be categorized into three groups as follows. The detailed explanation of each structural variable is also shown in Table 4.

- **Demographics:** Demographics information, including age and gender
- **Triage Information:** Information about patients upon their arrival at ED, including revisit, arrival by ambulance, is job related and triage level, *etc*.
- **Vital Signs:** Vital Signs information, including blood pressure, pulse, oxygen saturation, respiration and body temperature.
- **Outcome Variable:** There are two variables associated with our label. The first is “CPR”, where “1” indicates that the patient underwent CPR and “0” indicates that the patient did not. The second variable is “DEAD”, where “1” indicates the patient’s eventual death, and “0” indicates that the patient did not die.

The tabular data consists of 14 features, including demographics and triage information. Missing values in the tabular data are replaced with the mode across all patients. Categorical features are encoded using one-hot encoding.

##### Time series data

**Fig. 4:**
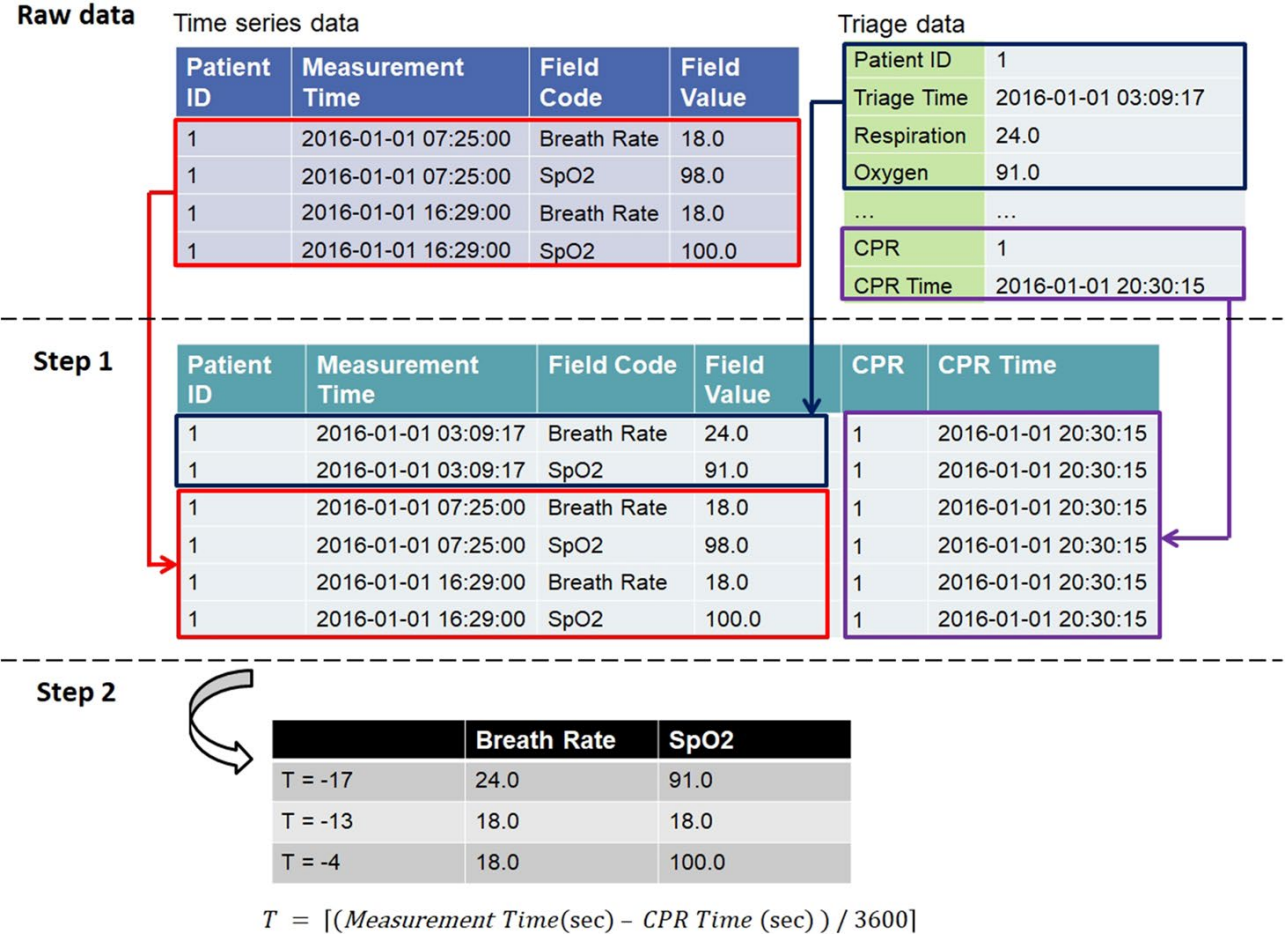
Data pre-processing, including integrating the raw data from both the time series data and the triage data, as well as arranging the data by time

The time series data consists of six continuous variables: systolic blood pressure (SBP), diastolic blood pressure (DBP), heart rate (HR), oxygen saturation (SPO2), body temperature (BT), and respiratory rate (RR). Below, we will outline how to preprocess the raw data from the database. We incorporated the time series data with the initial measurement from the triage data, as illustrated in step 1 of Fig. 4. Data merging was performed based on patient ID, incorporating CPR labels and CPR execution times from the triage data. Given that vital signs are manually recorded, there are occasional typo errors. Therefore, data cleaning is necessary. Any non-numeric characters become missing values upon converting full-width characters to half-width characters. Additionally, values falling outside the normal range for vital signs will be removed. The normal vital sign range is shown in Table 5.

After step 1 of Fig. 4, the subsequent step involves arranging the data by time. We used *T* to denote time. In cases where CPR equals 1, *T* is defined as:

1 $$\begin{aligned} T = \left\lceil {(Measurement Time(sec) - CPR Time (sec) ) / 3600}\right\rceil . \end{aligned}$$

where $\lceil \bullet \rceil$ refers to a ceiling function. In cases where CPR equals 0, *T* is defined as:

2 $$\begin{aligned} T = \left\lceil {(Measurement Time(sec) - Last Measurement Time (sec) ) / 3600)}\right\rceil . \end{aligned}$$

It is important to note that since our data only contains execution time of CPR rather than time of occurrence of CA of DNR samples, the DNR samples in our dataset are arranged such that the last measurement time was set as *T*=0. Therefore, the time when we predict the DNR sample is actually further away from the CA occurrence time.

**Table 4:** The detailed explanation of triage data

Variable names	Explanations
age	The age of the patient
sex	The gender of the patient
season	The season when the patient visit ED
revisit	Whether the patient revisit ED in 24 hours
is job related	Whether the patient visited ED because of work accident
department	The department of ED
day zone	Patient arrival time (1 for 07:00$\sim$15:00, 2 for 15:00$\sim$23:00, 3 for 23:00$\sim$07:00)
Arrival by ambulance	Whether the patient arrived by ambulance
major disease	Whether the patient has the IC card for severe illness
judgement code	The judgement code for describing the patient’s condition
triage	Five level of triage level in TTAS
pain index	Self evaluated pain score (0$\sim$10)
GCS	Glasgow Coma Scale of the patient
acute change	Any acute changes before entering ED
systolic	Systolic blood pressure
diastolic	Diastolic blood pressure
pulse	Pulse
oxygen	Oxygen saturation
respiration	Respiration
body temperature	Body temperature

**Table 5:** Normal range of the vital signs

	Normal range
SBP	[0, 300)
DBP	[0, 300)
HR	[0, 300)
SPO2	[0, 100]
BT	[0, 50)
RR	[0, 50)

#### Missing value imputation

We employed “Carrying the Most Recent Value Forward” (CF) methods for missing value imputation. With this approach, we can maintain the existing pattern of vital signs collection by nursing staffs without increasing their workload. We utilize our early warning system to predict whether a patient requires CPR every hour.

**Fig. 5:**
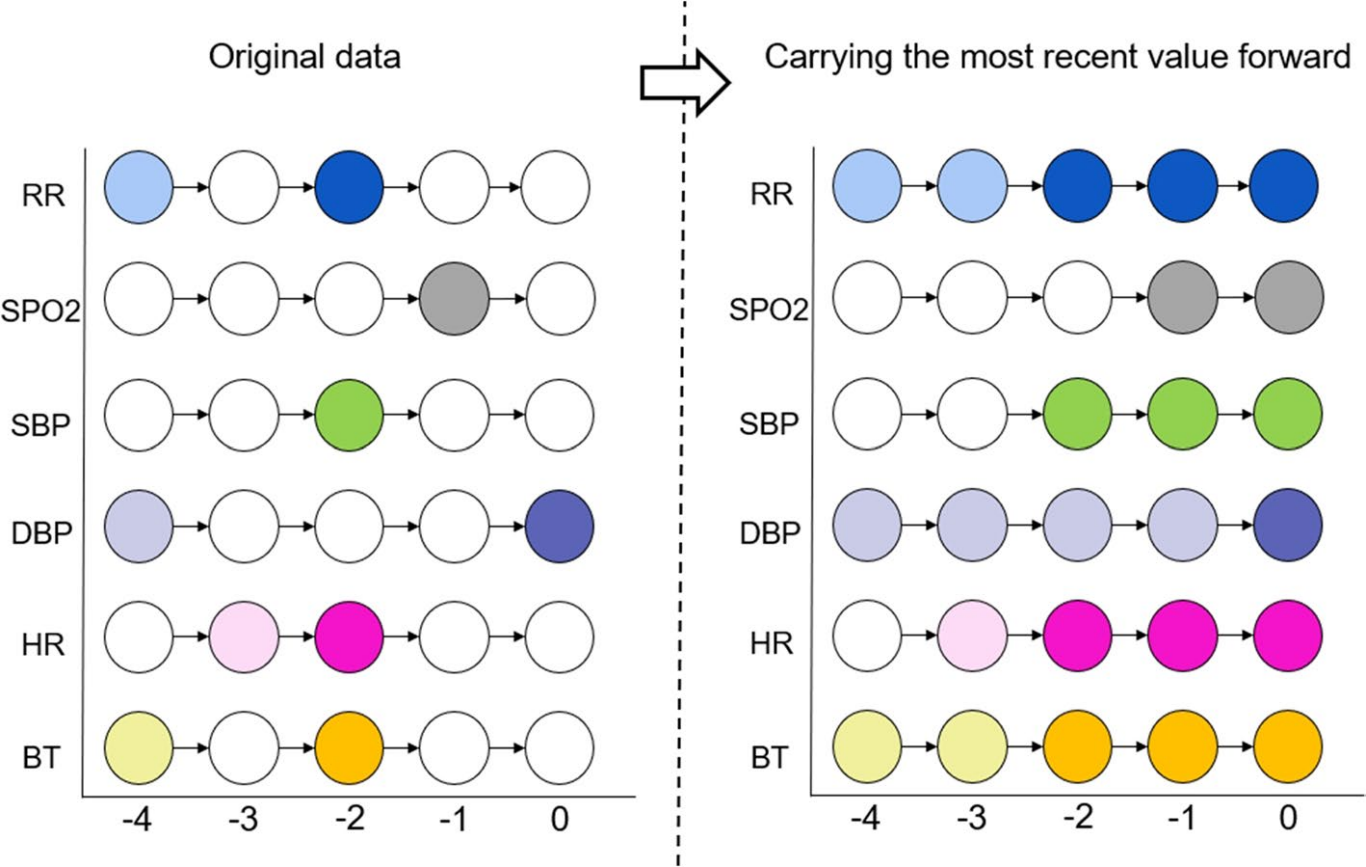
Missing data imputation for time-series data. The most recent data are carried forward

The left-hand side of Fig. 5 illustrates the original data in , where the horizontal axis represents time. The colored circles denote vital signs with recorded values, whereas the white circles represent missing values. Most missing values are imputed after applying the “carrying the most recent value forward” method. Missing values without past measurements as reference are filled using the median of that vital sign across all patients.

**Fig. 6:**
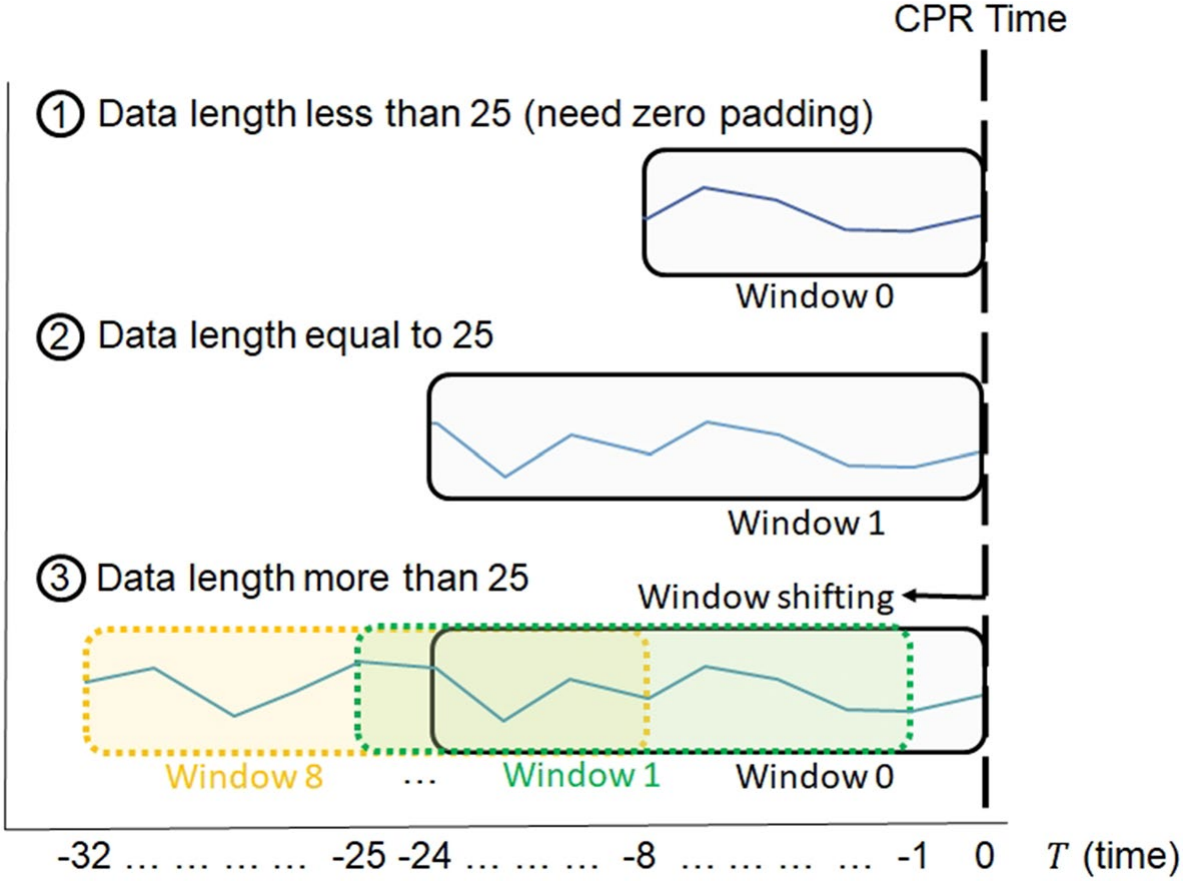
Three capture windows based on time-series data length. Three capture windows based on time-series data length

#### Data augmentation

After data pre-processing, the tabular data becomes a one-dimensional vector, and the time series data becomes an $n\times m$ matrix, where n denotes the number of vital signs, and m denotes the input sequence length. We will use a window from the sequence as the model’s input, and its window size is fixed at 25. As depicted in the Fig. 6, we aligned the window’s right end at $T=0$. We handled three cases based on the data length relative to the window size: 1).when the sequence length is less than 25, the window encompasses the entire sequence with zero padding; 2).when the data length equals 25, the window directly uses that sequence as input; and 3).when the sequence length exceeds 25, we use window shifting to increase the available data.

To address data imbalance, window shifting is applied only to the minority class. The window shifting stride is one hour. We will experiment with different window shifting maximum length to assess their impact on model performance. When the maximum length of the window shifting is *n*, the training data ranges from window 0 to window *n*. A larger value of *n* will facilitate the model in learning early prediction capabilities. However, determining how far the window shifts left from T=0 is a critical consideration. If windows sampled too far from the CPR execution time exhibit no significant differences compared to healthy instances, it could confuse the model’s discriminatory ability.

During the inference stage, we will test the model’s ability to early predict the need for CPR by inferring at different time points relative to the CPR execution time. In the experiment, we use window 0 from the testing data to evaluate the model’s predictive performance on all sequences. Additionally, we use window 8 from the testing data to assess the model’s ability to predict longer sequences eight hours in advance.

### Model design

In this section, we will introduce our model design. Our model primarily consists of four components, and each of which will be detailed in the following subsection.

#### The overall model architecture

**Fig. 7:**
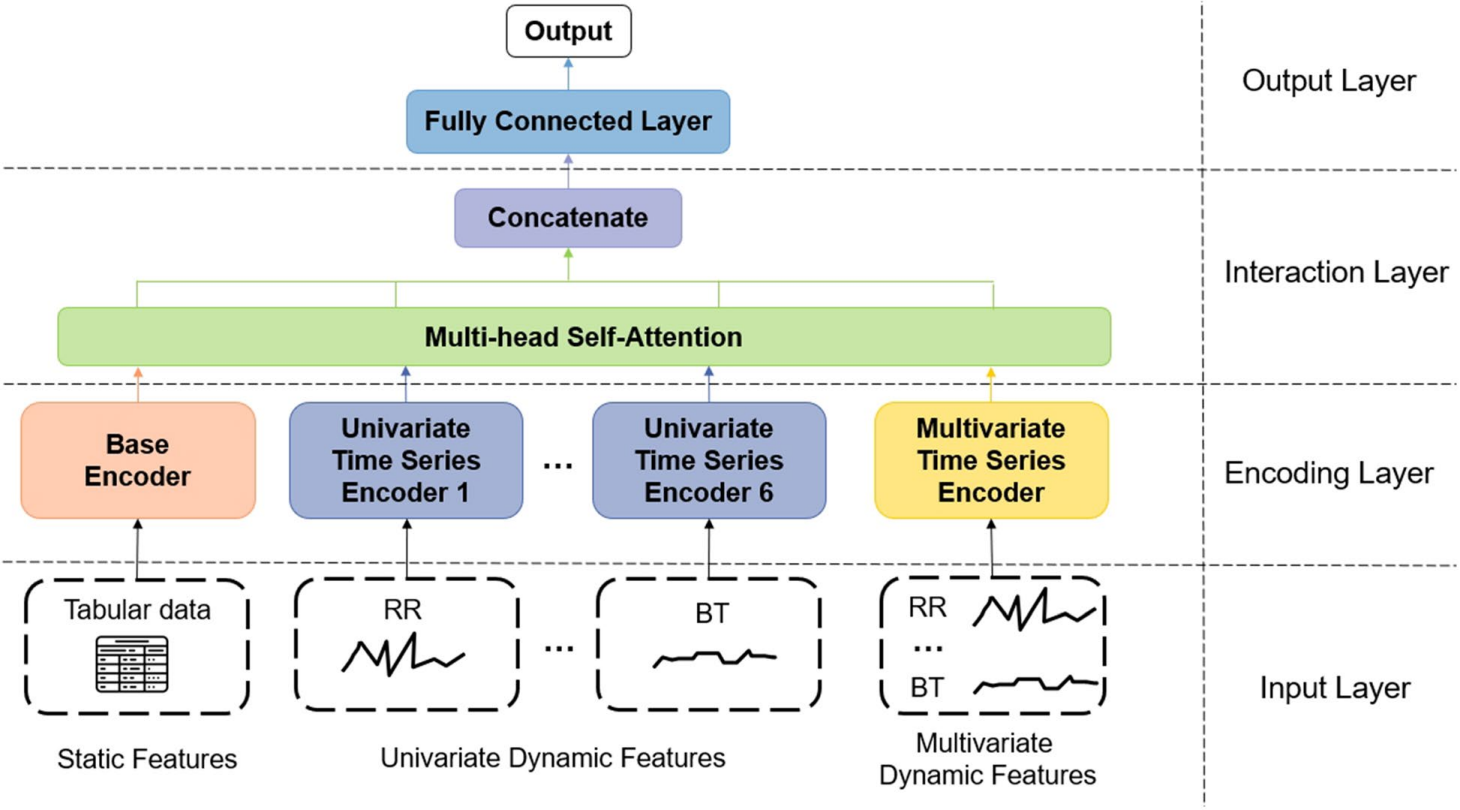
The overall model architecture. It consists of four components: the input layer, the encoding layer, the interaction layer, and the output layer

Our model architecture can be divided into four components: the input layer, the encoding layer, the interaction layer, and the output layer, as shown in Fig. 7. Firstly, the static features encompass the basic patient information, which refers to the tabular data, and the dynamic features pertain to the patient’s vital signs, which refers to the time series data. Both static and dynamic features are inputted into the input layer of the model, with the dynamic features being further divided into multivariate and six univariate.

Secondly, the static, univariate, and multivariate dynamic features are passed through distinct encoders: the “Base Encoder”, the “Univariate Time Series Encoder”, and the “Multivariate Time Series Encoder”, respectively, to extract representations.

Within the interaction layer, the representations learned by the encoders are then transformed into joint representations. The joint representations are concatenated and fed into the output layer to generate the final predictions. Further details about the encoding and interaction layers are described in the subsequent subsections.

#### The encoding layer

**Fig. 8:**
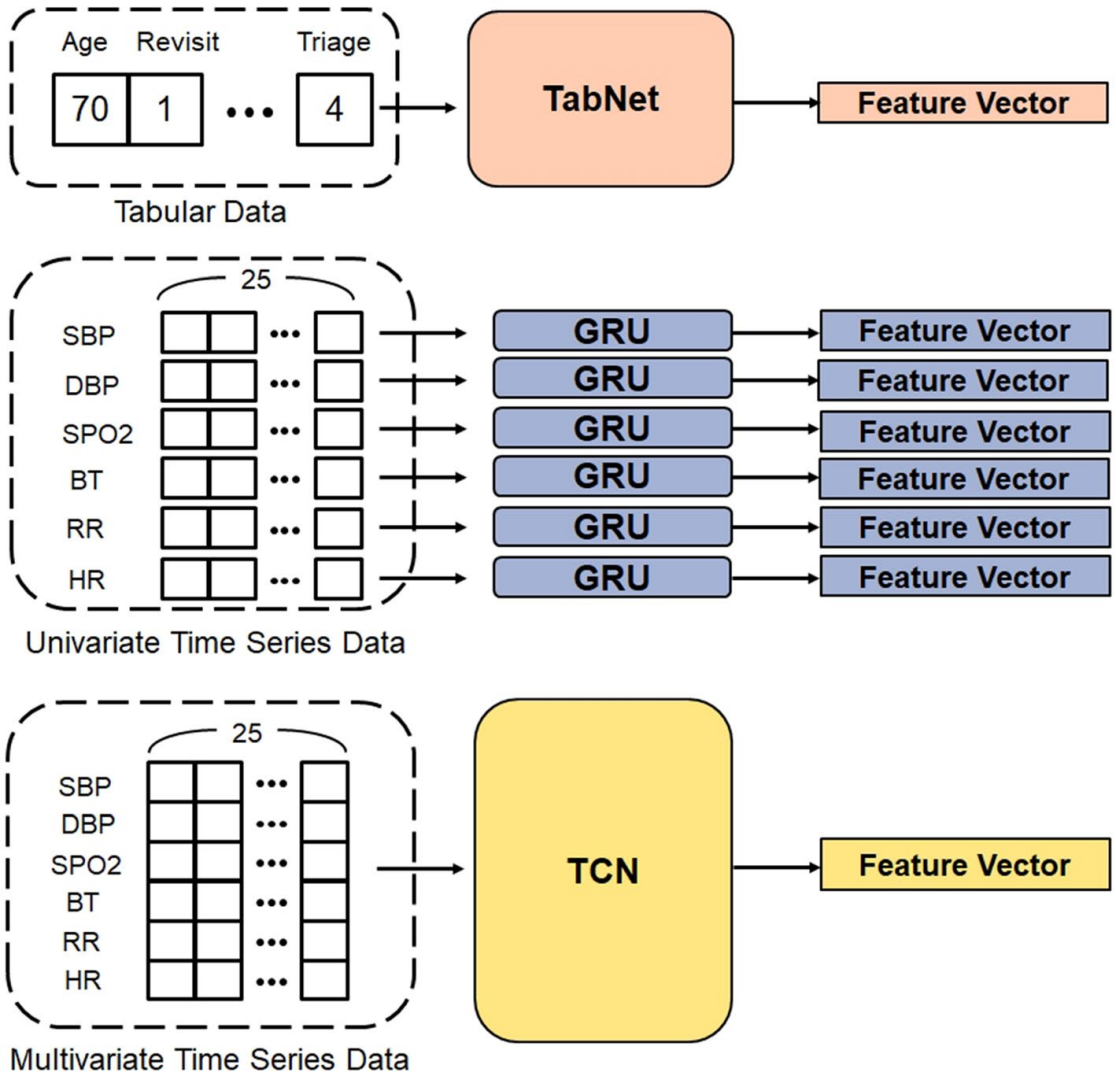
The components of the encoding layer. There are three encoders: Base Encoder, Univariate Time Series Encoder, and Multivariate Time Series Encoder

The encoding layer is responsible for transforming input data into a more meaningful representation. As shown in Fig. 8, there are three encoders, namely, Base Encoder, Univariate Time Series Encoder, and Multivariate Time Series Encoder.

##### Base encoder

The base encoder we chose is TabNet. TabNet [20] is a neural network model that resembles tree-based models, leveraging the strengths of both tree-based models and neural networks. The characteristics of TabNet enable our end-to-end model to engage in representation learning, facilitating the fusion of tabular and sequential data.

We have chosen TabNet as our backbone model for three main reasons.

First, traditional deep neural networks, such as stacked convolutional layers or multi-layer perceptrons (MLPs), are not well-suited for tabular data, as they tend to be significantly overparameterized. TabNet, on the other hand, combines the advantages of deep learning with traditional machine learning concepts, making it an ideal choice for handling tabular data.

Second, instead of relying on conventional MLP mechanisms, TabNet utilizes attention mechanisms and feature selection methods. This approach allows for a more comprehensive consideration of all features and helps capture the relationships between them.

Lastly, TabNet offers excellent compatibility. Compared to other deep learning models, it requires minimal data preprocessing and provides tabular data encoders, enabling us to conduct integrated training with other deep learning models.

Considering these advantages, we believe that TabNet is highly suitable for our data type and our need to collaborate with other models. Therefore, we have selected the TabNet encoder as our backbone model for processing tabular data.

The input dimensions in our setting are 32, while the output dimension is 8.

##### Time series encoder

Numerous research have introduced various deep learning architectures designed to manage temporal data, such as CNN [21], RNN [22], LSTM [23], and GRU [24]. In CA prediction, research has shown that TCN outperforms RNN-based models [19]. Therefore, our primary choice for the Time Series Encoder is TCN.

Despite the option to impute missing values in time series data using the previously mentioned method of “Carrying the Most Recent Value Forward”, using the median for imputation in time-series data results in a lack of significant variations in the trajectory of vital signs. This issue is most severe in variables such as blood oxygen, where the missing value rate reaches 0.815. In extreme cases, there are situations where patients have no recorded blood oxygen measurements at all. We suspect that feeding vital signs with significant trajectory changes and those without changes into the “Multivariate Time Series Encoder” may result in a representation of confused information. To address this concern, we have designed an approach incorporating the “Univariate Time Series Encoder” to extract features of vital signs individually. This ensures that vital signs with trajectory changes, processed through the “Univariate Time Series Encoder”, obtain a representation unaffected by other vital signs.

First, let us discuss the choice of “Multivariate Time Series Encoder”. Feeding all time series sequences into the encoder for encoding is common in the literature. Based on our experiments, TCN demonstrates the best performance; therefore, we have selected TCN as our multivariate encoder.

Next, we will discuss the “Univariate Time Series Encoder” selection. Including a “Univariate Time Series Encoder” in the model is intended to handle datasets with missing sequences. We also hope incorporating the “Univariate Time Series Encoder” will allow the model to gain richer information. Consequently, we chose GRU, which performs the second best, as our univariate encoder instead of TCN.

**Fig. 9:**
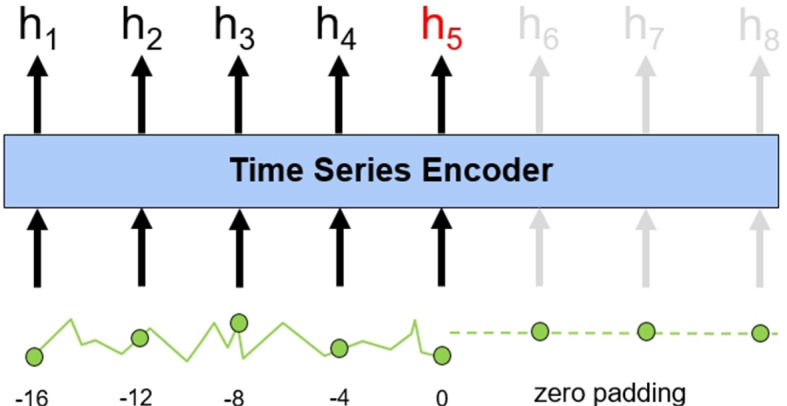
An example of modified zero padding. For short sequences, zero padding is performed at the end of time-series encoder

Considering that many emergency department patients do not stay for more than a day, zero-padding might negatively impact the model. Therefore, for sequences shorter than 25, we apply padding at the end of the sequence and obtained the output of the time series encoder earlier, as illustrated in Fig. 9.

#### The interaction layer

To capture implicit relations among representations from different encoders, the base features and multiple time series features are fed into a multi-head self-attention mechanism. Similar to the way how BERT [25] processes text, the representations outputs by the encoder layers can be seen as tokens and fed into the multi-head self-attention, enabling the capture of significant relationships between tokens to derive more crucial representations. Our multi-head self-attention mechanism employs four heads.

#### Loss function

The loss function used for training our model is Binary Cross Entropy, which is suitable for binary classification tasks. In a binary classification problem, we have a set of actual labels (usually 0 or 1), and the corresponding model predicts probabilities. Binary Cross Entropy measure the difference between actual labels and the corresponding model predicts probabilities. Its mathematical formula is shown as follows.

3 $$\begin{aligned} Loss_{Binary Cross Entropy}=-\frac{1}{N}\left[ \sum _{n=1}^{N}\left[ y_nlog(x_n) + (1-y_n)log(1-x_n) \right] \right] \end{aligned}$$

where *N* is the batch size, $y_n$ is the ground truth, and $x_n$ denotes the probability of the corresponding input.

## Results and discussion

### Experimental setup

The detailed system environment is outlined in Table 6. We utilized PyTorch 1.12.1 and Python 3.8.13 to run all algorithms on a computer equipped with Nvidia RTX 3080, Intel Core i5-8400K, and 16GB RAM. Throughout the training process, we employed the Adam optimizer [26] with a learning rate of $10^{-4}$ and a batch size of 64. Early stopping was implemented when there was no improvement in validation AUPRC for 80 consecutive epochs.

**Table 6:** The details of the system environment

System environment	
OS	Windows 10
CPU	i5-8400K
RAM	16GB
GPU	Nvidia RTX 3080
Framework	Python 3.8.13 with PyTorch 1.12.1

**Fig. 10:**
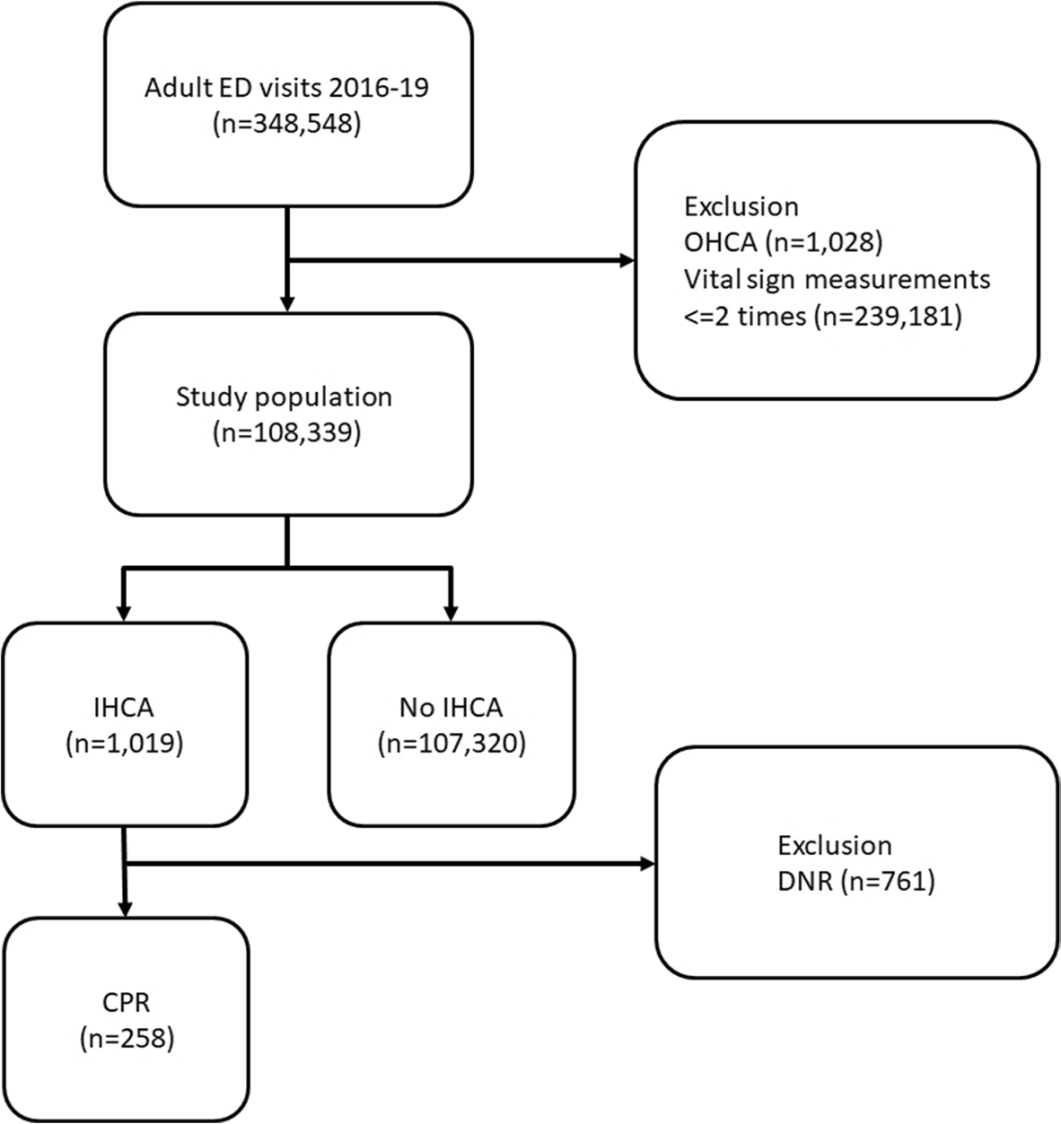
Data flowchart. Abbreviations: ED = Emergency Department. OHCA = Out-of-Hospital Cardiac Arrest. IHCA = In-Hospital Cardiac Arrest. DNR = do not resuscitate. CPR = cardiopulmonary resuscitation

### Dataset

The study population comprises ED visits of patients from 2016 to 2019. Exclusions are made for out-of-hospital cardiac arrest (OHCA) patients and those with fewer than two vital signs measurements. The NTUH-CA dataset encompasses 108,339 patients (1,019 CA and 107,320 No CA). The NTUH-CPR dataset, excluding samples with do-not-resuscitate(DNR), contains 107,578 patients (258 CPR and 107,320 No CPR). The data filtering process is illustrated in Fig. 10.

**Fig. 11:**
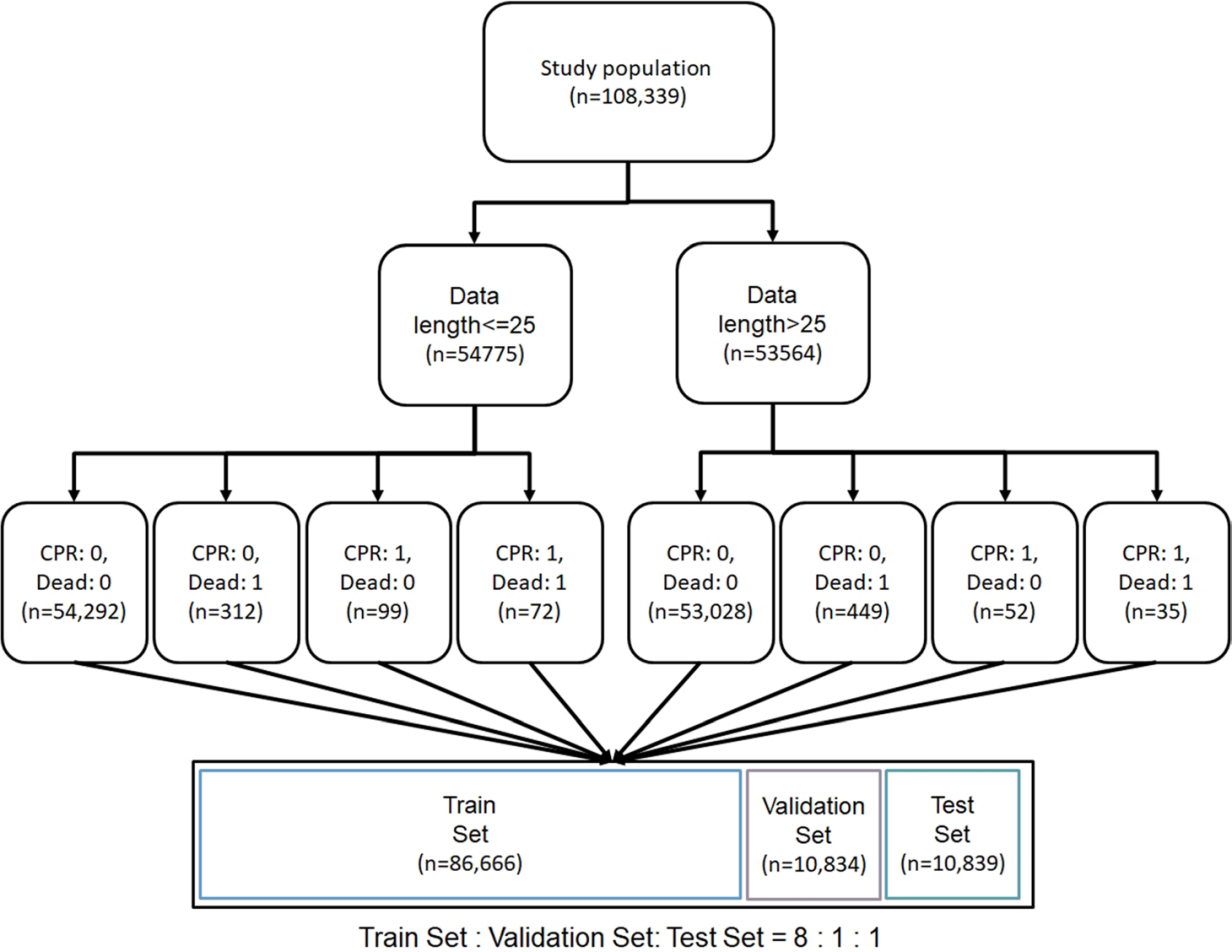
Data split process. Using stratified sampling, the data is first divided into long and short data length sets. Then, it is further separated by class (CPR, Dead) to ensure that the data length distribution and class distribution in the training set, validation set, and test set are approximately similar

**Table 7:** Dataset stastics

	Train set	Validation set	Test set
Non-event, n (%)	85853 (99.1)	10733 (99.1)	10734 (99.0)
CA, n (%)	813 (0.9)	101 (0.9)	105 (1.0)
CPR, n (%)	205 (0.2)	25 (0.2)	28 (0.3)

We first divide the data into short and long time segments to ensure the distribution balance between short and long time series data across the training, validation, and test sets. Secondly, to ensure consistent class distribution among the three sets, we categorize the data into four combinations: (CPR:0, Dead:0), (CPR:0, Dead:1), (CPR:1, Dead:0), and (CPR:1, Dead:1). Subsequently, we split them into train, validation, and test sets in an 8:1:1 ratio. The process of data split is depicted in Fig. 11. The label distribution across the three sets is presented in Table 7.

**Fig. 12:**
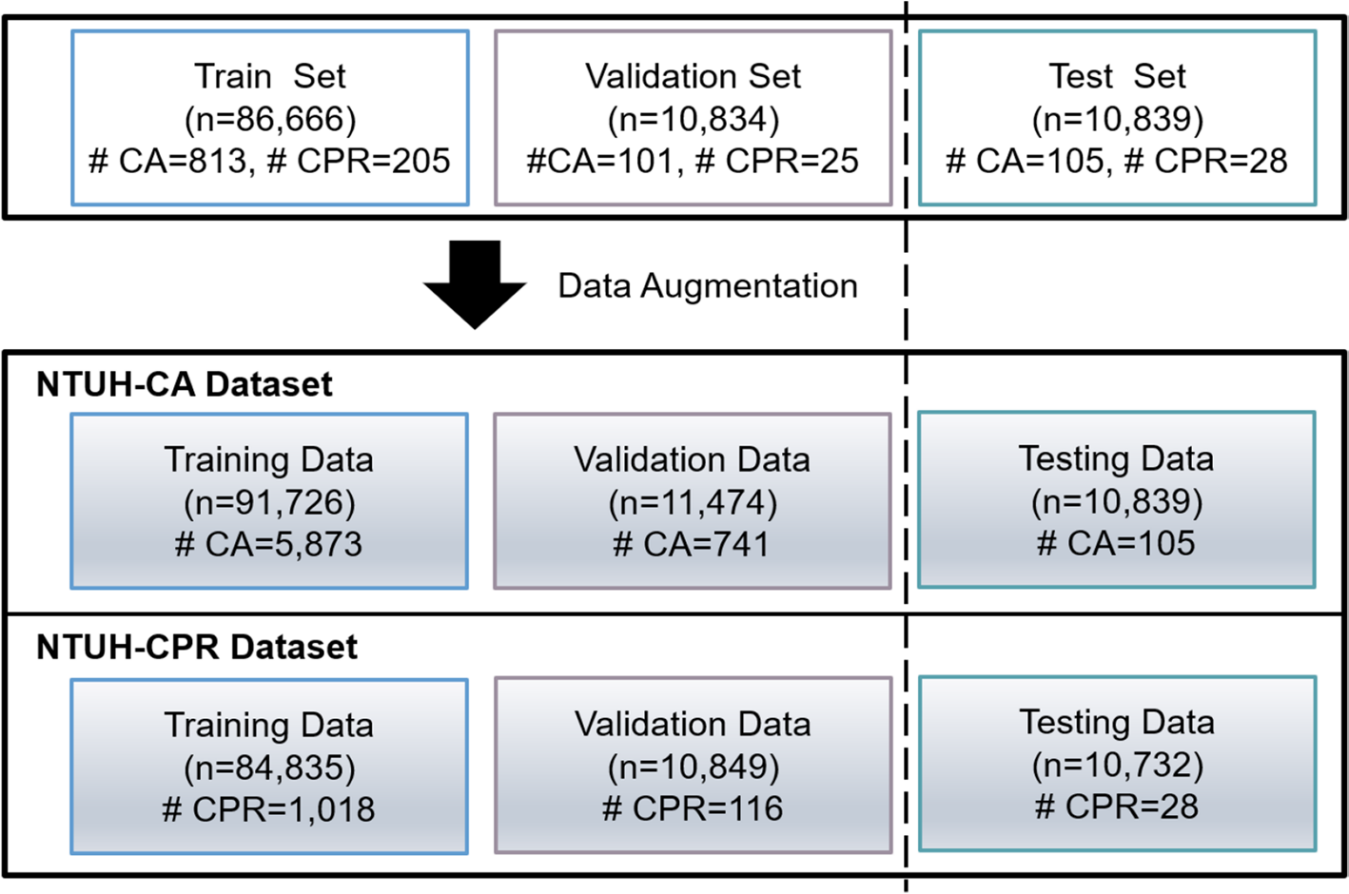
The dataset after data augmentation

Data augmentation is performed for the train and validation sets, as mentioned in “Data augmentation” section. The number of CA samples in the training data increases by approximately sixfold, whereas the number of CPR samples increases by approximately fourfold. The augmented data volume is illustrated in Fig. 12.

### Evaluation procedure

We will evaluate our model using the testing data from the NTUH-CA and NTUH-CPR datasets. We will compare our system with the clinical early warning score. The following explanation is illustrated using CPR samples, which also applies to DNR samples.

We will test the model’s predictions within one hour before CPR occurrence and eight hours before CPR occurrence-the prediction within one hour before CPR occurrence is tested on all samples in the testing data. In comparison, the number of positive samples will be fewer for predictions eight hours before CPR occurrence. Positive samples refer to CPR samples that match the window length of eight hours before the occurrence of CPR.

In the following experiments, we will present the model’s performance on the testing data. Evaluation metrics include recall, precision, F1-score, the area under the precision-recall curve (AUPRC), and the area under the receiver operating characteristic curve (AUROC).

### NTUH-CA dataset

We conducted experiments to evaluate our model, as discussed in “Missing value imputation” section on the NTUH-CA dataset. In this section, we compare different imputation methods. Moreover, we compared our system against the existing EWS. The ablation study demonstrated the performance of different window shifting maximum length and individual components of the model. Finally, we experimented using the model trained on the NTUH-CA dataset to predict CPR outcomes directly.

**Table 8:** The effectiveness of different time series model

Model	Time to CA	AUPRC	AUROC	Recall	Precision	F1-score
LSTM	0 hr	0.2600	0.9026	0.7333	0.0858	0.1537
GRU	0 hr	0.3084	0.9175	**0.7810**	0.0877	0.1577
TCN	0 hr	**0.3535**	**0.9286**	0.7333	**0.0905**	**0.1611**
LSTM	8 hrs	0.1984	0.9218	0.7174	0.0387	0.0734
GRU	8 hrs	0.1905	**0.9257**	**0.7391**	0.0383	0.0729
TCN	8 hrs	**0.2719**	0.9162	0.7174	**0.0409**	**0.0774**

*Abbreviations*: *LSTM* Long Short-Term Memory, *GRU* Gated Recurrent Unit, *TCN* Temporal Convolutional Network, *CA* Cardiac Arrest, *AUPRC* the area under precision recall curve, *AUROC* the area under the receiver operating characteristic curve

#### Evaluation of time series model

Table 8 display the performance of standard time series models on the NTUH-CA dataset. The results showed that TCN [27] achieved an AUPRC of 0.3294, outperforming GRU [24] and LSTM [23], and attained an AUPRC of 0.2025 in the early prediction.

**Table 9:** The results of prediction within 1 hour before the occurrence of CA

Research studies	Dataset	AUPRC	AUROC	Recall	Precision	F1-score
MEWS [5]	NTUH-CA	0.2357	0.8812	0.7048	0.1393	0.1576
NEWS [6]	NTUH-CA	0.3668	0.9229	0.7143	0.1391	0.2329
EDICAS [7]	NTUH-CA	0.4042	0.9205	0.7238	0.1201	0.2060
LR	NTUH-CA	0.3122	0.8809	0.6190	0.1157	0.1949
Deep EDICAS	NTUH-CA	**0.5178**	**0.9388**	0.7143	**0.1553**	**0.2551**
Jang et al. [14]	Private	-	0.936	**0.939**	0.045	0.086

*Abbreviations*: *LR* Logistic Regression, *NEWS* National Early Warning Score, *MEWS* Modified Early Warning Score, *EDICAS* Emergency Department In-hospital Cardiac Arrest Score, *CA* Cardiac Arrest, *AUPRC* the area under precision recall curve, *AUROC* the area under the receiver operating characteristic curve

**Table 10:** The results of prediction made eight hours before the occurrence of CA

Research studies	Dataset	AUPRC	AUROC	Recall	Precision	F1-score
MEWS [5]	NTUH-CA	0.0992	0.8394	0.5000	0.0294	0.0555
NEWS [6]	NTUH-CA	0.1276	0.8649	0.4783	0.0453	0.0827
EDICAS [7]	NTUH-CA	0.0768	0.9002	0.5652	0.0446	0.0827
LR	NTUH-CA	0.1118	0.8597	0.6087	0.0529	0.0974
Deep EDICAS	NTUH-CA	**0.2798**	**0.9046**	**0.7826**	**0.0811**	**0.1479**

*LR* Logistic Regression, *NEWS* National Early Warning Score, *MEWS* Modified Early Warning Score, *EDICAS* Emergency Department In-hospital Cardiac Arrest Score, *CA* Cardiac Arrest, *AUPRC* the area under precision recall curve, *AUROC* the area under the receiver operating characteristic curve

**Fig. 13:**
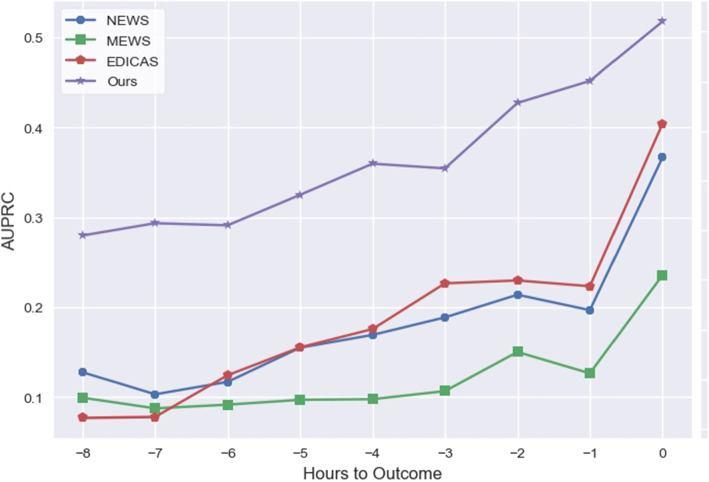
Model Performance over time. The graph shows AUPRC in hours prior to cardiac arrest. Abbreviation: AUPRC = the area under precision recall curve

**Fig. 14:**
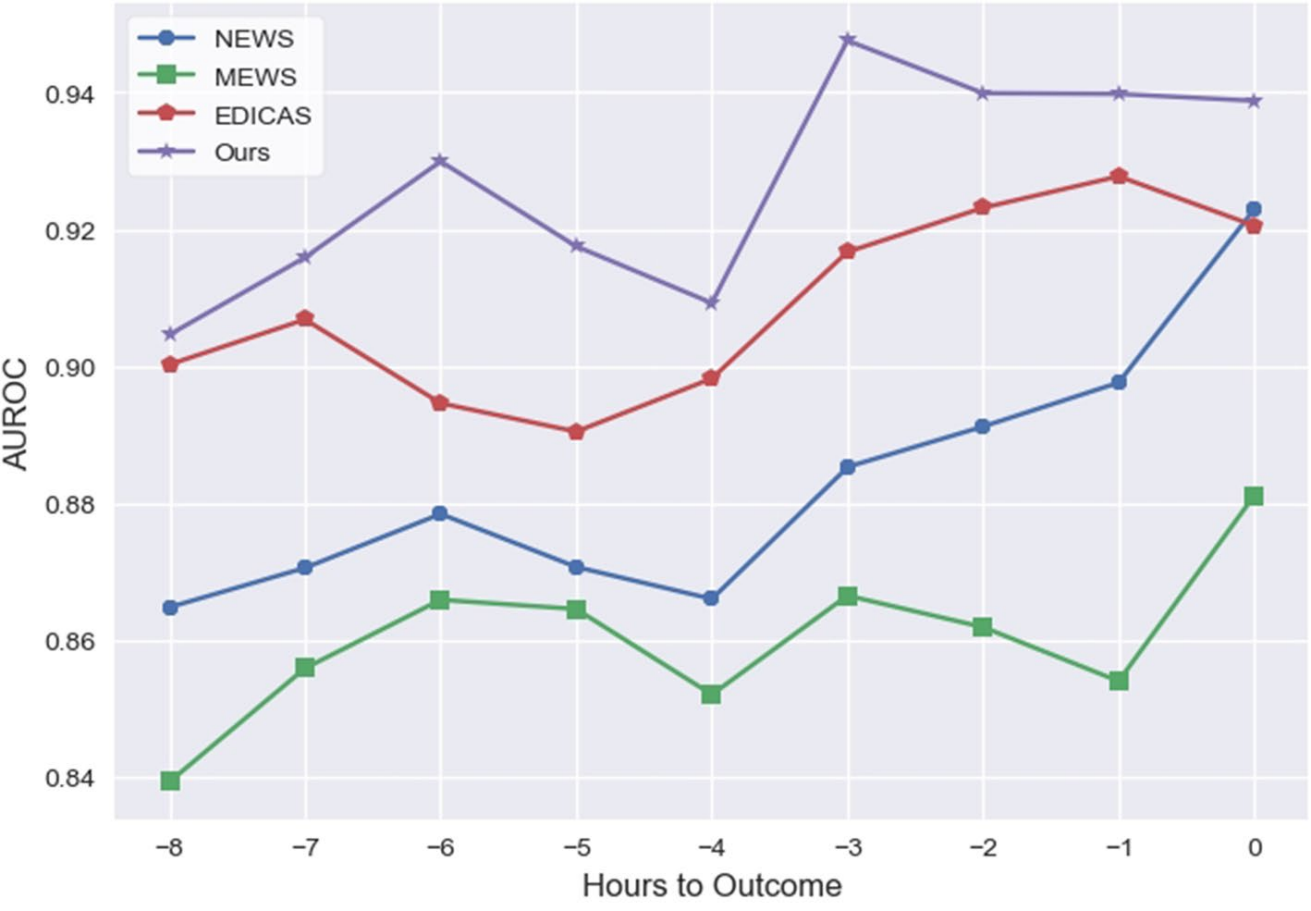
Model Performance over time. The graph shows AUROC in hours prior to cardiac arrest. Abbreviation: AUROC = the area under the receiver operating characteristic curve

#### Compared with other EWS

Our early warning system achieved an AUPRC of 0.5178 and an AUROC of 0.9388, surpassing the Early Warning Scores (EWS) and logistic regression, as shown in Table 9. Additionally, in making predictions eight hours in advance, our system excelled with an AUPRC of 0.2798 and an AUROC of 0.9046, as indicated in Table 10. Figures 13 and 14 respectively present the AUPRC and AUROC performances for different time predictions.

#### Ablation studies

##### Effectiveness of different window shifting maximum length

We experimented with window shifting maximum length of 13-hour, 8-hour, and 0-hour to assess their impact on model performance. Table 11 indicates that 0-hour achieved the best performance with an AUPRC of 0.5756 for predictions within one hour before CA occurrence. However, for predictions eight hours before CA occurrence, window shifting maximum length of 13-hour demonstrated the best performance across all evaluation metrics. Therefore, a window shifting maximum length of 13-hour provides better early prediction capabilities and is the optimal choice.

##### Effectiveness of different architectures

We examined the impact of each component on the overall model performance. Base Encoder, Univariate Time Series Encoder, Multivariate Time Series Encoder, and Multi-head self-attention were individually removed from our model to evaluate their effectiveness. Table 12 reveals that removing each component results in a performance decline. In terms of the extent of performance decline, the components affecting the model performance the most are, in sequence, Base Encoder, Univariate Time Series Encoder, Multi-head self-attention, and Multivariate Time Series Encoder.

### NTUH-CPR dataset

After the experimental analysis of the NTUH-CA dataset, we now turn our attention to the NTUH-CPR dataset.

**Table 11:** The effectiveness of different window shifting maximum length

Window shifting maximum length	Time to CA	AUPRC	AUROC	Recall	Precision	F1-score
13 hrs	0 hr	0.5178	**0.9388**	0.7143	**0.1553**	**0.2551**
8 hrs	0 hr	0.4537	0.9198	0.7714	0.1006	0.1780
0 hr	0 hr	**0.5756**	0.9382	**0.8762**	0.0978	0.1759
13 hrs	8 hrs	**0.2798**	**0.9046**	**0.7826**	**0.0811**	**0.1479**
8 hrs	8 hrs	0.1766	0.8757	0.6739	0.0411	0.0774
0 hr	8 hrs	0.1336	0.8301	0.6087	0.0319	0.0607

*Abbreviations*: *CA* Cardiac Arrest, *AUPRC* the area under precision recall curve, *AUROC* the area under the receiver operating characteristic curve

**Table 12:** The effectiveness of different modalities

Model	Time to CA	AUPRC	AUROC	Recall	Precision	F1-score
**Full**	0 hr	**0.5178**	**0.9388**	0.7143	**0.1553**	**0.2551**
−Base encoder	0 hr	0.3257	0.8955	0.7429	0.0659	0.1211
−Univariate time series encoder	0 hr	0.3589	0.9138	0.7333	0.0890	0.1588
−Multivariate time series encoder	0 hr	0.4829	0.9291	**0.8190**	0.0734	0.1348
−Multi-head self attention	0 hr	0.4424	0.9186	0.7143	0.1090	0.1892
**Full**	8 hrs	**0.2798**	0.9046	** 0.7826**	**0.0811**	**0.1479**
−Base encoder	8 hrs	0.1690	0.9135	0.7609	0.0307	0.0590
−Univariate time series encoder	8 hrs	0.1870	0.9138	0.6522	0.0367	0.0694
−Multivariate time series encoder	8 hrs	0.2381	**0.9423**	**0.7826**	0.0321	0.0617
−Multi-head self attention	8 hrs	0.1987	0.8937	0.6957	0.0496	0.0926

*Abbreviations*: *CA* Cardiac Arrest, *AUPRC* the area under precision recall curve, *AUROC* the area under the receiver operating characteristic curve

#### Compared with other EWS

**Fig. 15:**
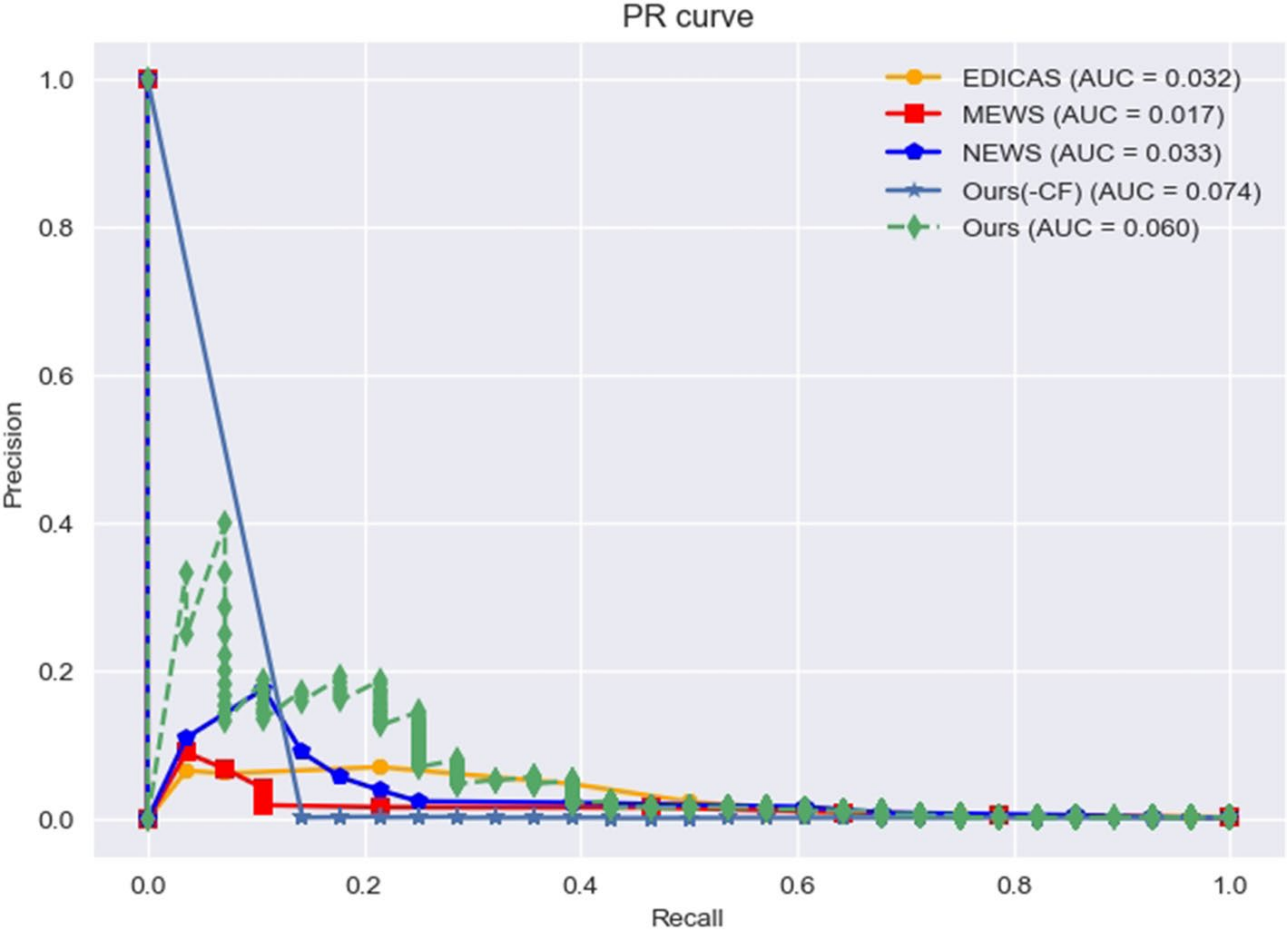
Precision-recall curves of different early warning scores on NTUH-CPR dataset. Abbreviation: NTUH-CPR = National Taiwan University Hospital-cardiopulmonary resuscitation

Table 13 demonstrates that our system achieved an AUPRC of 0.0604, surpassing the existing EWS. Surprisingly, removing the Base Encoder (BE) resulted in a higher AUPRC of 0.0741. The PR Curve is shown in Fig. 15. From the figure, it can be observed that our system achieved higher precision performance only when a recall is below 0.4, while the area under the curve (AUPRC) of our system, excluding BE, mostly comes from recalls below 0.2. It is observed that the precision is particularly low, leading to a high false alarm rate, making it unsuitable for practical clinical applications. Therefore, models optimized based on AUPRC may have issues that are not suitable for clinical use. It is worth discussing whether there are new validation metrics that simultaneously consider AUPRC, AUROC, Recall, and Precision. Additionally, our model’s performance significantly decreases in making prediction eight hours before CPR occurrence (Table 14). We will discuss the relationship between window shifting maximum length and early prediction capabilities in the ablation study.

**Table 13:** The results of prediction within 1 hour before the occurrence of CPR

Research studies	Dataset	AUPRC	AUROC	Recall	Precision	F1-score
MEWS [5]	NTUH-CPR	0.0172	0.7855	0.4643	0.0168	0.0325
NEWS [6]	NTUH-CPR	0.0334	0.8263	0.3929	0.0232	0.0437
EDICAS [7]	NTUH-CPR	0.0322	**0.8333**	0.5000	0.0245	0.0467
Deep EDICAS	NTUH-CPR	0.0604	0.7438	0.3929	**0.0451**	**0.0809**
Deep EDICAS(-BE)	NTUH-CPR	**0.0741**	0.5216	**0.6071**	0.0027	0.0054

*Abbreviations*: *NEWS* National Early Warning Score, *MEWS* Modified Early Warning Score, *EDICAS* Emergency Department In-hospital Cardiac Arrest Score, *BE* Base Encoder, *AUPRC* the area under precision recall curve, *AUROC* the area under the receiver operating characteristic curve

**Table 14:** The results of prediction made eight hours before the occurrence of CPR

Research studies	Dataset	AUPRC	AUROC	Recall	Precision	F1-score
MEWS [5]	NTUH-CPR	0.0082	0.6999	0.2857	0.0026	0.0052
NEWS [6]	NTUH-CPR	0.0065	0.7782	0.1429	0.0022	0.0042
EDICAS [7]	NTUH-CPR	0.0028	0.7917	0.2857	**0.0036**	**0.0071**
Deep EDICAS	NTUH-CPR	0.0007	0.5209	0.0000	0.0000	0.0000
Deep EDICAS(-BE)	NTUH-CPR	**0.1444**	**0.8142**	**1.0000**	0.0011	0.0022

*Abbreviations*: *NEWS* National Early Warning Score, *MEWS* Modified Early Warning Score, *EDICAS* Emergency Department In-hospital Cardiac Arrest Score, *BE* Base Encoder, *AUPRC* the area under precision recall curve, *AUROC* the area under the receiver operating characteristic curve

#### Ablation studies

##### Effectiveness of different window shifting maximum length

We investigated the impact of window shifting maximum length (12-hour, 8-hour, and 0-hour) on model performance. Table 15 shows that the 8-hour window shifting maximum length outperformed the 12-hour in both scenarios of making predictions one hour before CPR occurrence and eight hours before CPR occurrence. While increasing the window shifting maximum length to 8-hour enhanced the model’s early prediction capability compared to a 0-hour, it led to a decrease in predictive performance for the hour just before CPR. How to make the model simultaneously excel in predicting short sequences and early predicting long sequences remain an unresolved challenge.

##### Effectiveness of different architectures

In this section, we experimented with the impact of different components on our model using the NTUH-CPR dataset. The model’s performance with the Base Encoder, Univariate Time Series Encoder, Multi-head self-attention, and Multivariate Time Series Encoder removed is shown in Table 16. We observed a decrease in performance after removing the Univariate Time Series Encoder, Multi-head self-attention, and Multivariate Time Series Encoder. Although removing the Base Encoder increased AUPRC, the model tended to predict 1, leading to the lowest precision. Therefore, the overall model’s performance is more stable with the complete set of components.

### Interpretability

**Fig. 16:**
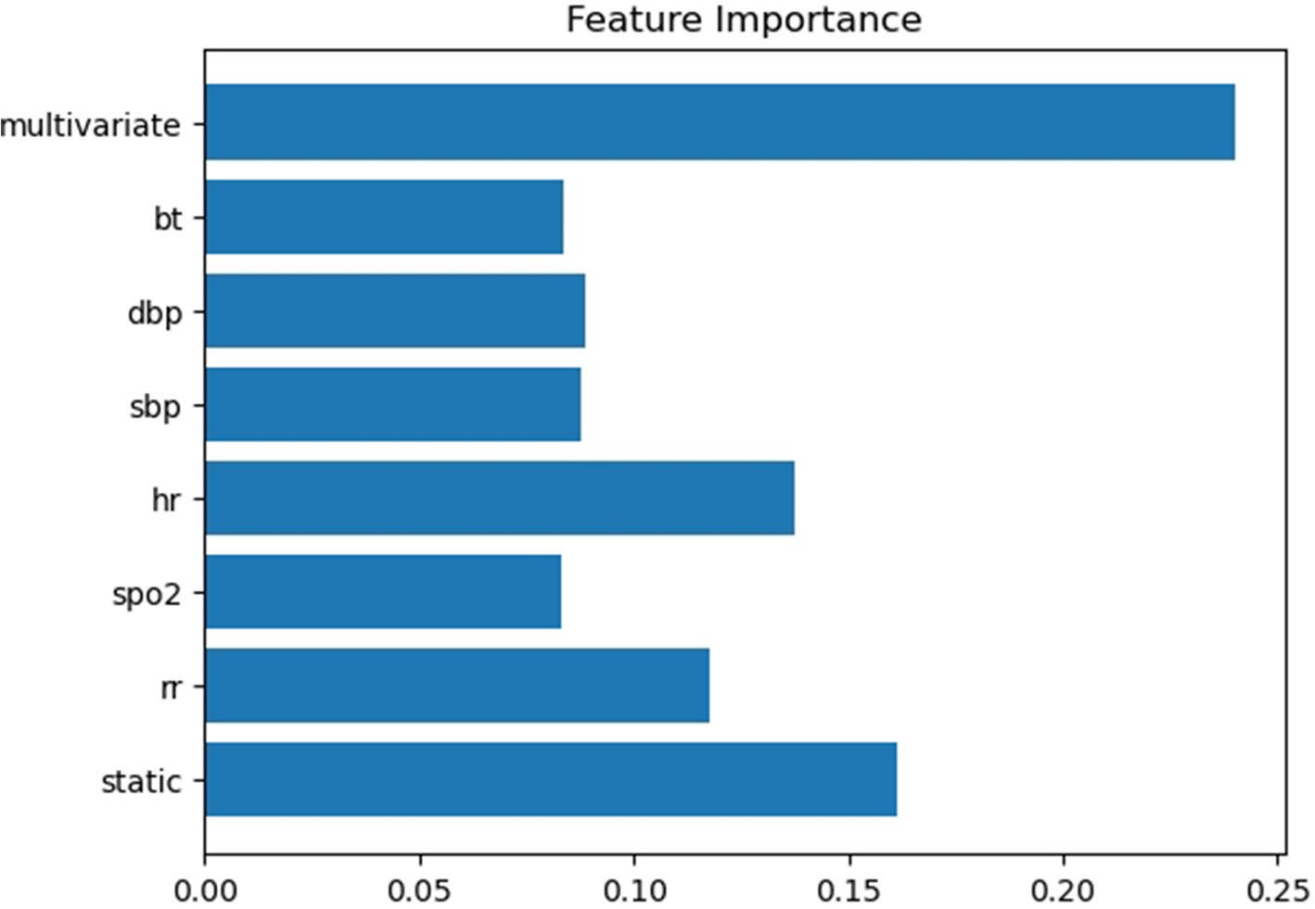
Features importance among positive samples

The attention scores of the Multi-head Self-attention revealed which features the model focuse on. Figure 16 illustrate the feature importance for all CA patients in the testing data. As expected, the multivariate time series feature emerge as the most critical one. When examining individual vital signs, among the six vital signs, heart rate (hr) was identified as the most important, followed by respiratory rate (rr) as the second most crucial one.

**Table 15:** The effectiveness of different window shifting maximum length

Window shifting maximum length	Time to CPR	AUPRC	AUROC	Recall	Precision	F1-score
12 hrs	0 hr	0.0091	0.7672	0.2500	0.0154	0.0289
8 hrs	0 hr	0.0140	**0.7902**	**0.4286**	0.0207	0.0395
0 hr	0 hr	**0.0604**	0.7438	0.3929	**0.0451**	**0.0809**
12 hrs	8 hrs	0.0010	0.5555	0.1429	0.0022	0.0044
8 hrs	8 hrs	**0.0023**	**0.8235**	**0.2857**	**0.0035**	**0.0069**
0 hr	8 hrs	0.0007	0.5209	0.0000	0.0000	0.0000

*AUPRC* the area under precision recall curve, *AUROC* the area under the receiver operating characteristic curve

**Table 16:** The effectiveness of different modalities

Model	Time to CPR	AUPRC	AUROC	Recall	Precision	F1-score
**Full**	0 hr	0.0140	**0.7902**	0.4286	**0.0207**	**0.0395**
−Base encoder	0 hr	**0.0741**	0.5216	**0.6071**	0.0027	0.0054
−Univariate time series encoder	0 hr	0.0070	0.7684	0.2143	0.0074	0.0143
−Multivariate time series encoder	0 hr	0.0094	0.7691	0.2143	0.0071	0.0137
−Multi-head self attention	0 hr	0.0116	0.7685	0.2143	0.0086	0.0165
**Full**	8 hrs	0.0023	**0.8235**	0.2857	**0.0035**	**0.0069**
−Base encoder	8 hrs	**0.1444**	0.8142	**1.0000**	0.0011	0.0022
−Univariate time series encoder	8 hrs	0.0016	0.5607	0.2857	0.0025	0.0049
−Multivariate time series encoder	8 hrs	0.0008	0.5307	0.1429	0.0012	0.0024
−Multi-head self attention	8 hrs	0.0012	0.5396	0.1429	0.0014	0.0029

*Abbreviations*: *AUPRC* the area under precision recall curve, *AUROC* the area under the receiver operating characteristic curve

For clinical applications, it will be helpful for healthcare providers to explore more detailed feature importance in multivariate time series in the future work.

**Fig. 17:**
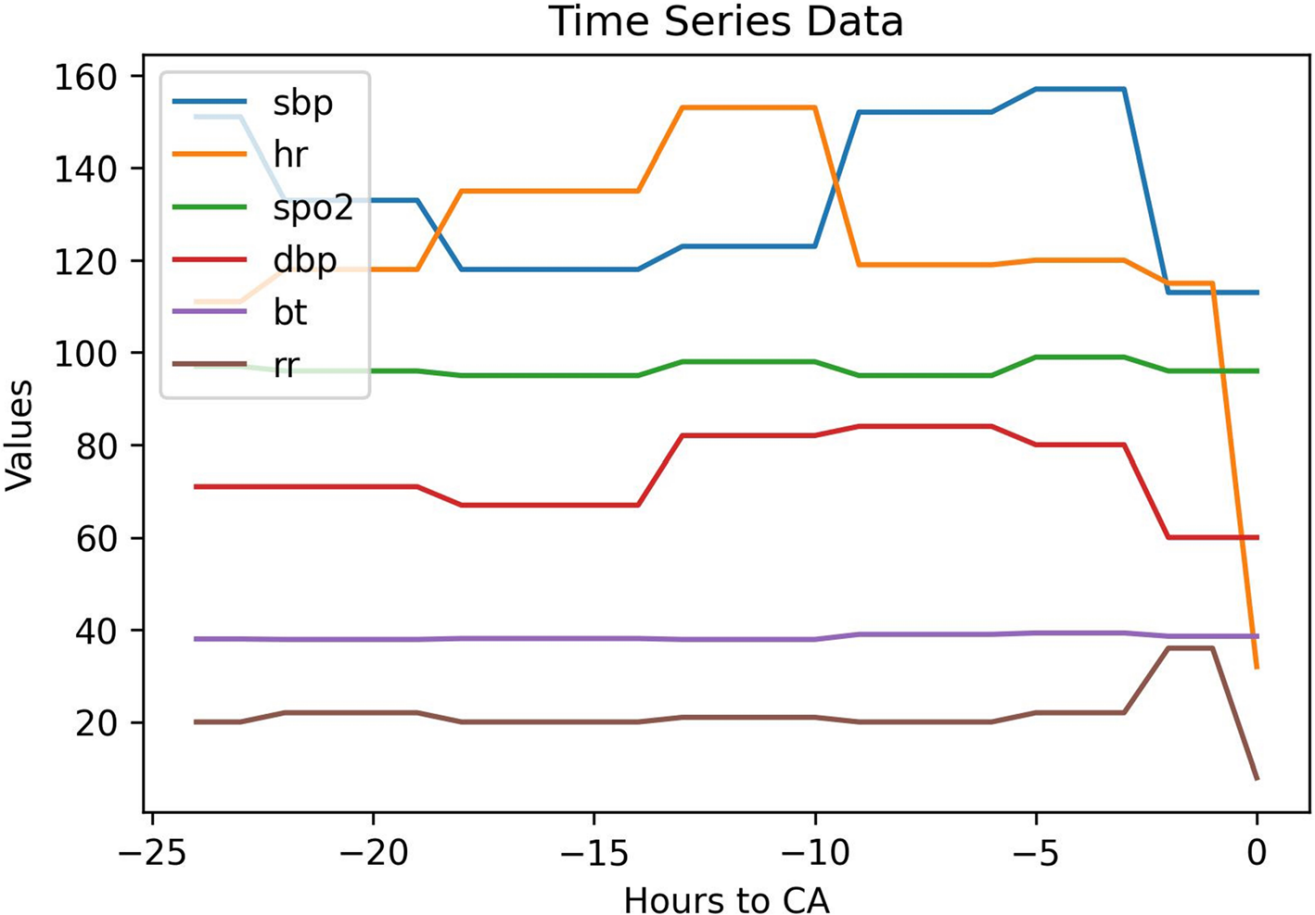
The time-series data of a real patient’s vital signs over time

**Fig. 18:**
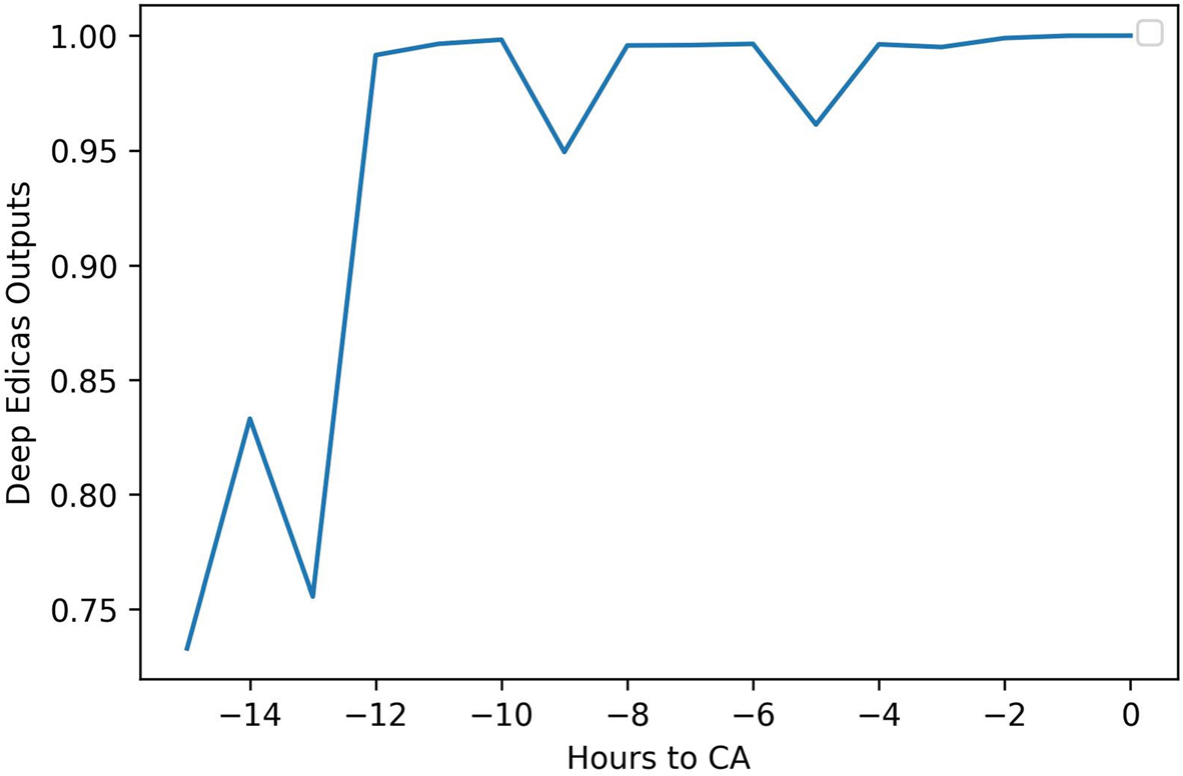
The model’s predictions of the real patient over time

To illustrate the system’s predictions and clinical progression, we selected a real patient from our data. Figure 17 shows the time series data of the real patient’s changes over time, while Fig. 18 displays the model’s predicted output over time. This 93-year-old man presented to our emergency department with respiratory distress and pneumonia symptoms. Approximately 12 hours preceding the cardiac arrest, he started to have an increased heart rate and elevated diastolic blood pressure. Over time, this patient eventually progressed to cardiac arrest with dropped heart rates and respirations, suggesting cardiac and respiratory failure.

## Limitations

Our study has some limitations. First, rare CA events (i.e., highly imbalanced data) resulted in low precision when evaluating our model. The occurrence of positive samples in NTUH-CA dataset was quite rare, with a base rate of 0.0097 in the test set (Table 7). Our model’s precision was 0.1553 (Table 9), suggesting that the positive predictive rate can be improved by 16 times. In other words, clinicians would be much more likely to find a true CA event. Given the high stakes of CA events, clinicians in our research group found this level of precision acceptable. Nonetheless, the model would need future rounds of improvement to further increase its precision to lessen the burden of false alarms posed on frontline medical staff. Second, We recognize that transformers are popular in deep learning. It is indeed worth investigating whether transformers can also demonstrate their excellent performance in time series data, and this will be a direction we will continue to pursue in our future research.

## Conclusion

ED overcrowding is one of the most severe public health issues globally, and one of its serious complications is CA. This is a highly destructive and high-risk event for patients, families, and healthcare professionals. An early detection system for CA is crucial, but the clinically adopted EWS struggles to achieve high sensitivity and a low false alarm rate simultaneously. There is limited research on applying deep learning to emergency room data, especially for patients with short stays, and little experimentation has been conducted on the early prediction capabilities of these deep models as far as we know. Therefore, applying deep learning to an Early Warning System for CA is still a worthwhile area of research. Our research aims to design a Deep Early Warning System that can potentially integrate with existing hospital systems, addressing challenges such as irregular sampling rates, frequent missing values, severe data imbalance, and a lack of interpretability.

In this paper, we designed a hybrid model where the Base Encoder (Tabnet) extracted information from tabular data, the Multivariate Time Series Encoder (TCN) extracted from time series data (six vital signs), and six Univariate Time Series Encoders (GRU) extracted information from individual physiological data. Finally, the fusion representation through Multi-head Self-attention was obtained.

Our research was conducted on two datasets: the NTUH-CA and the NTUH-CPR datasets. Our system achieved an AUPRC of 0.5178 and an AUROC of 0.9388 on the NTUH-CA dataset, showing an improvement of 0.1136 and 0.0183 over EDICAS in AUPRC and AUROC. For early prediction, our system achieved an AUPRC of 0.2798 and an AUROC of 0.9046, demonstrating superiority over other EWS. Furthermore, a 13-hour window shifting maximum length on the NTUH-CA dataset address data imbalance and enhance the model’s early prediction capability. The ablation study demonstrated that each component of our model contributed to its effectiveness.

However, in the more challenging CPR prediction task (unexpected deterioration with few measurements prior to CPR event), our system had yet to outperform other EWS on the NTUH-CPR dataset consistently. The experimental results with different window shifting maximum lengths indicated that increasing the window shifting maximum length improved the model’s early prediction capability but sacrificed the ability to predict short sequences. Developing a system that combines the ability to predict short sequences and early prediction for long sequences is a direction for future research.

Future work will involve adjusting the primary optimization metric, such as an improved version of AUPRC. Areas under the recall curve can be weighted, with higher weights assigned to areas where recall exceeds 0.6, ensuring that the model achieves a basic level of recall performance. Additionally, for the Univariate Time Series Model, we can explore incorporating time differences as features, allowing the model inputs to be flexible regarding time units, which can reduce a significant amount of missing data. Furthermore, experimenting with training the model using only window 8 (vital signs from $T=0$ to $T=-32$) might lead to better early prediction performance, although challenges related to reduced data volume need to be addressed. Finally, using a transformer as a time series encoder is also a key area for future research. Clinically, implementing the Deep EDICAS in the ED would be warranted to test its real-world effectiveness.

## Data Availability

The datasets used and/or analyzed during the current study are available from the corresponding author on reasonable request.

## Abbreviations

ED: Emergency Department
CA: Cardiac Arrest
IHCA: In-Hospital Cardiac Arrest
EWS: Early Warning Scores
NEWS: National Early Warning Scores
MEWS: Modified Early Warning Scores
DEWS: Deep learning-based early warning system
EDICAS: Emergency Department In-hospital Cardiac Arrest Score
DL: Deep Learning
CPR: Cardiopulmonary Resuscitation
DNR: Do-not-resuscitate
NTUH: National Taiwan University Hospital
SBP: Systolic blood pressure
DBP: Diastolic blood pressure
HR: Heart rate
SPO2: Oxygen saturation
BT: Body temperature
RR: Respiratory rate
CF: Carrying the Most Recent Value Forward
BE: Base Encoder
AUROC: the area under the receiver operating characteristic curve
AUPRC: the area under precision recall curve
